# Bioluminescence Goes Dark: Boosting the Performance of Bioluminescent Sensor Proteins Using Complementation Inhibitors

**DOI:** 10.1021/acssensors.2c01726

**Published:** 2022-11-30

**Authors:** Alexander Gräwe, Maarten Merkx

**Affiliations:** Laboratory of Protein Engineering, Department of Biomedical Engineering and Institute for Complex Molecular Systems, Eindhoven University of Technology, Eindhoven 5600 MB, The Netherlands

**Keywords:** biosensors, split luciferase, protein engineering, antibodies, autoinhibition, protein switches

## Abstract

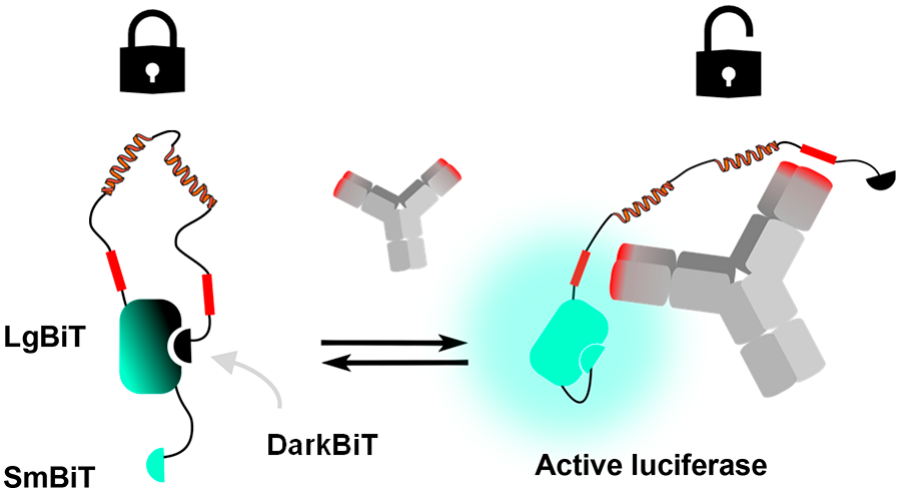

Bioluminescent sensor proteins have recently gained popularity in both basic research and point-of-care diagnostics. Sensor proteins based on intramolecular complementation of split NanoLuc are particularly attractive because their intrinsic modular design enables for systematic tuning of sensor properties. Here we show how the sensitivity of these sensors can be enhanced by the introduction of catalytically inactive variants of the small SmBiT subunit (DarkBiTs) as intramolecular inhibitors. Starting from previously developed bioluminescent antibody sensor proteins (LUMABS), we developed single component, biomolecular switches with a strongly reduced background signal for the detection of three clinically relevant antibodies, anti-HIV1-p17, cetuximab (CTX), and an RSV neutralizing antibody (101F). These new dark-LUMABS sensors showed 5–13-fold increases in sensitivity which translated into lower limits of detection. The use of DarkBiTs as competitive intramolecular inhibitor domains is not limited to the LUMABS sensor family and might be used to boost the performance of other bioluminescent sensor proteins based on split luciferase complementation.

Current standardized immunoassays such as ELISA and related technologies allow for sensitive and specific detection of a wide range of analytes, but the need for multiple wash and incubation steps limits their use in point-of-care (POC) applications. Lateral flow immunoassays (LFIAs) have been introduced as an inexpensive, fast and user-friendly alternative that allows optical analyte detection without specialized equipment.^1^ However, LFIAs are less suited for quantitative measurements and they do not reach the sensitivity of classical, laboratory-based immunoassays.^2^ An alternative approach to the heterogeneous assay principle employed in ELISA and LFIA is provided by homogeneous immunoassay formats that allow single-step quantitative detection of analytes directly in solution. Here bioluminescence has proven particularly attractive. Unlike fluorescence, whose sensitivity is limited by background fluorescence and light scattering, bioluminescence does not require light excitation, resulting in a high signal-to-background ratio that is easily detected even in complex media such as blood plasma.^3−5^

Recent years have witnessed the development of a range of new bioluminescent sensor platforms for homogeneous analyte detection.^6^ Most of these employ NanoLuc, a small and stable engineered luciferase that produces bright and stable blue luminescence by converting furimazine to furimamide.^3^ Two mechanisms for the modulation of bioluminescent sensor output are widely employed. In BRET-based sensor proteins, analyte binding induces a conformational change that modulates the distance and thus affects the efficiency of energy transfer between a NanoLuc donor and a fluorescent acceptor. An important advantage of these BRET-based sensor proteins is that they allow for ratiometric detection, which renders them independent of sensor concentration and much less sensitive to substrate depletion and differences in environmental conditions.^7^ However, developing sensors with a large change in BRET, and thus a high sensitivity, can be challenging as it requires conformational control of both the OFF- and ON-state of the sensor.

Successful examples include the LUCID sensors, which employ competitive displacement of an intramolecular ligand analogue to detect small molecule analytes,^8,9^ and the **Lum**inescent **A**nti**b**ody **S**ensor (LUMABS) proteins for antibody detection.^10,11^ The LUMABS proteins switch from a high BRET state, in which a NanoLuc domain and a green fluorescent acceptor are held in close proximity by two helper domains, to a low BRET state in which the distance between NanoLuc and its acceptor is increased due to binding of the target antibody to recognition motifs at each end of the semiflexible linker in the sensor (Figure 1A). While the modular nature of the LUMABS design allows it to be applied to detect any antibody, the nature of the recognition motifs still affect the change in emission ratio and thus the sensors sensitivity.^4,11^

**Figure 1:**
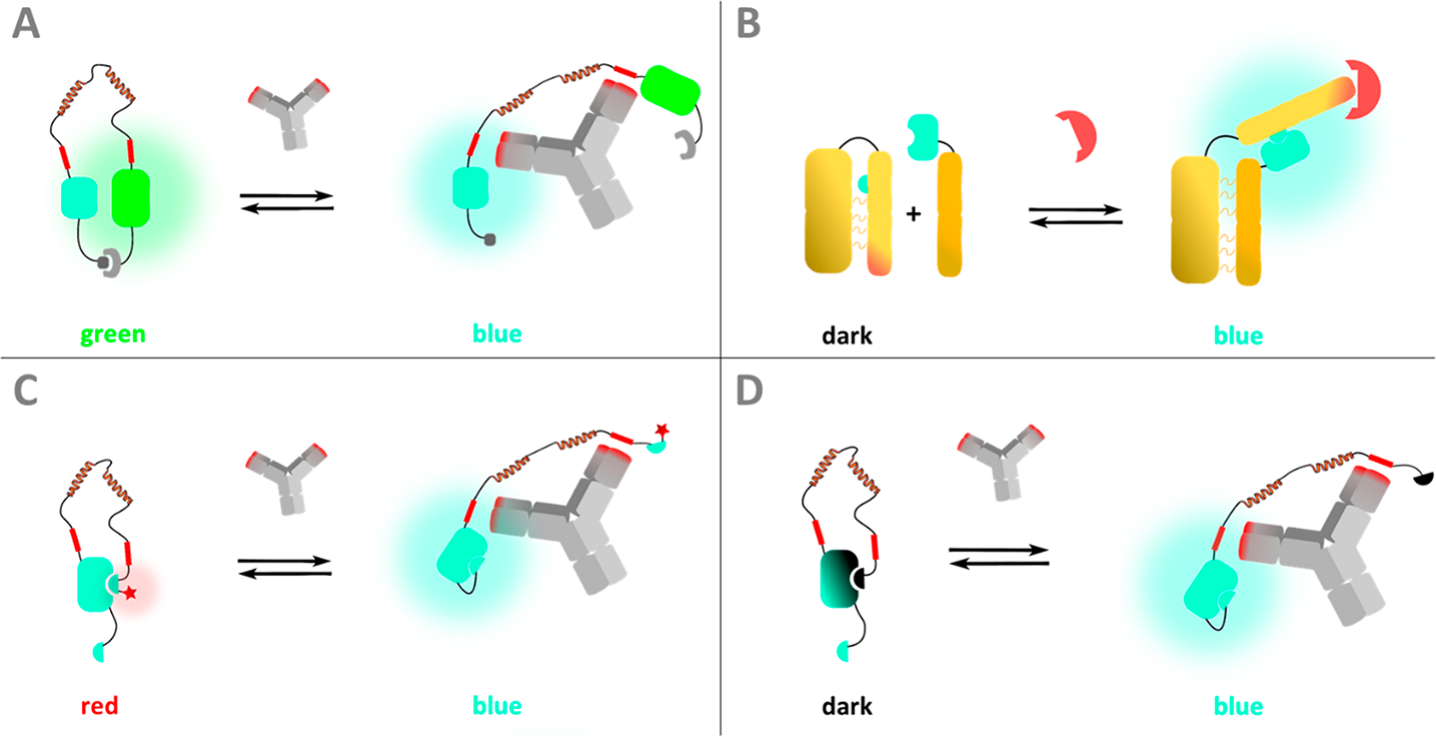
NanoLuc and NanoBiT based biosensor concepts. (A) BRET-based antibody sensors (LUMABS) using full-length NanoLuc and fluorescent BRET acceptor with additional helper domains. (B) LucCage switch with shielded SmBiT in the OFF state and thermodynamically controlled ON state. (C) NB-LUMABS with NanoBiT, using two SmBiTs with different affinities and a coupled dye (Cy3). (D) dark-LUMABS concept. A catalytically inactive SmBiT is spatially separated from LgBiT upon target binding, allowing complementation of LgBiT by a lower affinity, active SmBiT.

A second design strategy for bioluminescent sensors uses a split version of NanoLuc (NanoBiT) and depends on analyte-dependent control of intramolecular split NanoLuc complementation. NanoBiT consists of a large protein domain (LgBiT, 18 kDa) and a smaller peptide domain (SmBiT, ≈1.3 kDa), whose affinity can be systematically tuned between *K*_D_ = 0.7 nM and 190 μM. A recent example are the so-called lucCage sensors developed by the Baker group.^13,14^ In this two component sensor system a target binding motif and SmBiT are fused to a “latch” domain that binds intramolecularly to a cage domain, effectively preventing SmBiT to bind to the LgBiT containing sensor component. Binding of the target analyte by the binding motif disrupts the interaction between the latch domain and the cage domain, enabling complementation of SmBiT with LgBiT and an increase in blue bioluminescence intensity (Figure 1B). In addition to their intrinsically modular design, NanoBiT and split-NanoLuc sensors typically show a larger increase in sensor response compared to BRET systems.^3,5^ However, lucCage and most other NanoBiT sensors are intensiometric, requiring additional calibration steps to compensate for matrix effects and time-dependent substrate turnover.

Recently our group reported a design strategy to render sensors based on NanoBiT complementation ratiometric by employing a design in which LgBiT can interact with two competing SmBiT domains, of which one contained a Cy3 fluorescent acceptor domain.^12,15^ This design is reminiscent of the alternate frame folding concept originally introduced by the Loh group.^16^ Application of the NanoBiT concept for antibody detection yielded so-called NB-LUMABS sensors with a ratiometric response that switched between red emission in the absence of antibody and blue emission in the presence of antibody (Figure 1C).^12^ While the larger spectral separation between red and blue emission resulted in an increased change in emission ratio, the sensitivity of these sensor systems was still smaller than that typical of intensiometric sensors based on NanoBiT.

Here, we establish competitive complementation of LgBiT with a catalytically active SmBiT and a catalytically inactive SmBiT (DarkBiT) as a novel and modular design approach to increase the dynamic range of bioluminescent sensor proteins (Figure 1D). We first established the affinity of the inactivated SmBiT before integrating it in the NB-LUMABS design. Screening for optimal intramolecular affinities between the LgBiT, active SmBiT, and DarkBiT components yielded highly improved antibody sensors for three different antibodies that target linear, cyclic, and discontinuous epitopes, respectively. We also demonstrate how the intensiometric dark-LUMABS sensors can be combined with green enhanced Nanolantern (GeNL)^17^ as a calibrator luciferase to allow ratiometric detection while retaining a high sensitivity.

## Results and Discussion

### Determination of DarkBiT Affinity

**Figure 2:**
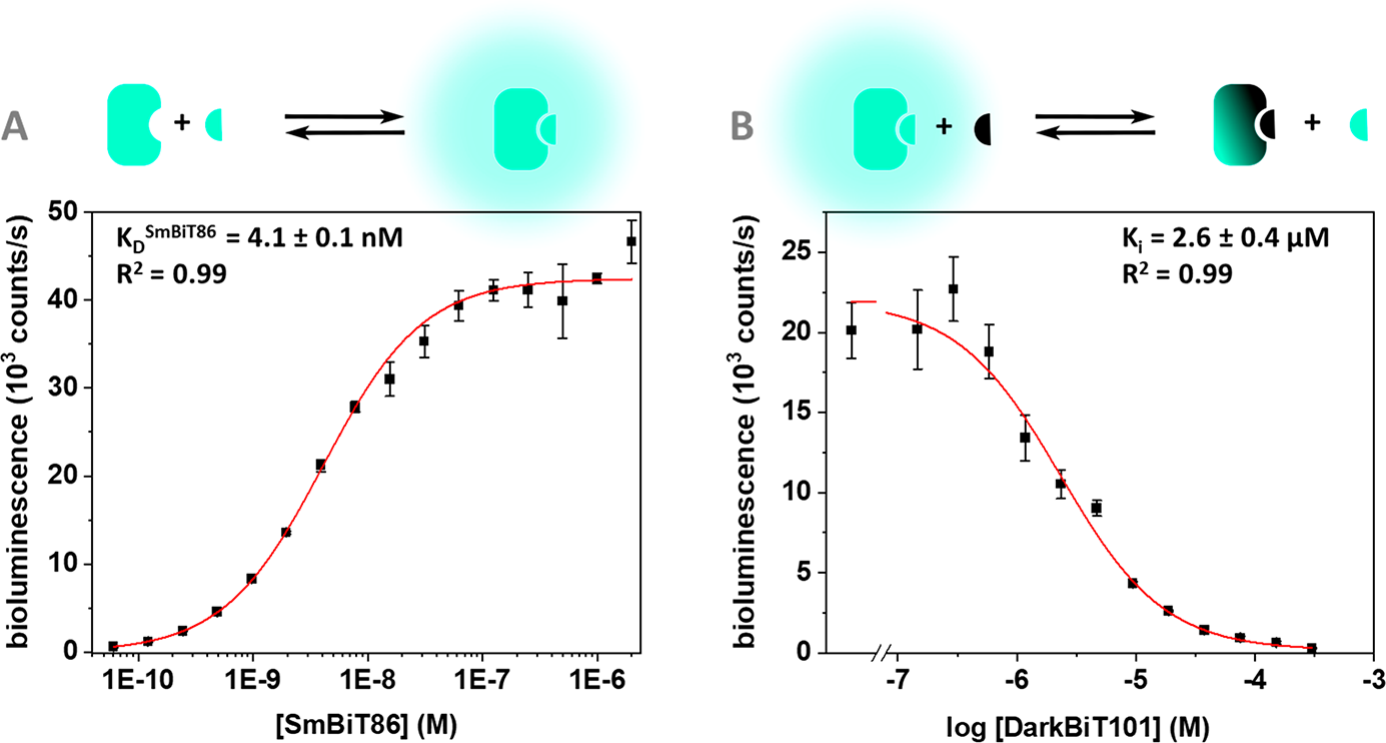
DarkBiT binding characterization. (A) Titration to determine SmBiT86 affinity for LgBiT. Experimental conditions: 1×PBS, 0.02% Tween20, 15 pM LgBiT, incubation for 2 h at 20 °C before addition of 1:1000 diluted NanoGlo. (B) Competition experiment to determine *K*_i_ of DarkBiT101 (*K*_D_^DarkBiT^). Experimental conditions: 1×PBS, 0.02% Tween20, 75 pM SmBiT86, 0.75 nM LgBiT, incubation at 4 °C for 16 h before addition of 1:1000 NanoGlo. Tween20 was used as supplement instead of BSA as BSA affected the intensity of the luminescent signal and decreased the apparent affinity in titration experiments of SmBiT peptide binding to LgBiT (Figure S1). Error bars represent the standard deviation based on 3 technical replicates.

To effectively suppress background complementation of LgBiT by SmBiT variants, catalytically inactive SmBiT variants were employed. These “dark” peptides or DarkBiTs have the same sequence as their corresponding SmBiT variants except for a single R > A mutation that renders them catalytically inactive. A DarkBiT fused to GST was previously used to reduce the background signal in membrane translocation studies.^18,19^ Since the relative affinities of SmBiTs and DarkBiTs are important design parameters for the construction of protein switches, we first established whether the R > A mutation affects the binding to LgBiT. As complementation of a DarkBiT with LgBiT cannot be directly assessed using bioluminescence, a competition experiment was performed in which the affinity of a DarkBiT peptide was derived from its ability to inhibit the formation of a catalytically active complex between LgBiT and SmBiT86, a high affinity variant that has been reported to have a low nanomolar affinity.^20,21^ We first established the *K*_D_ of the latter interaction in a direct titration experiment, yielding *K*_D_^SmBiT86^ = 4.1 ± 0.1 nM (Figure 2A). Next, we performed a competition experiment in which DarkBiT101 (sequence VTGYALFEKES) was titrated to a fixed concentration of LgBiT and SmBiT86, leading to reduced bioluminescence at higher DarkBiT101 concentrations. A fit of the titration curve yielded a *K*_i_^DarkBiT^ of 2.6 ± 0.4 μM (for calculations, see the Supporting Information), which is very similar to the affinity of the parent, catalytically active SmBiT101 (reported *K*_D_ = 2.5 μM) (Figure 2B).^20^ These results show that, perhaps surprisingly, the R > A mutation does not affect the binding affinity between the small and the large subunit. We therefore assumed that the *K*_D_’s of DarkBiT variants are similar to the published affinities of their corresponding SmBiT peptides.^20^

### Intramolecular Autoinhibition in LgBiT-DarkBiT Fusion Proteins

**Figure 3:**
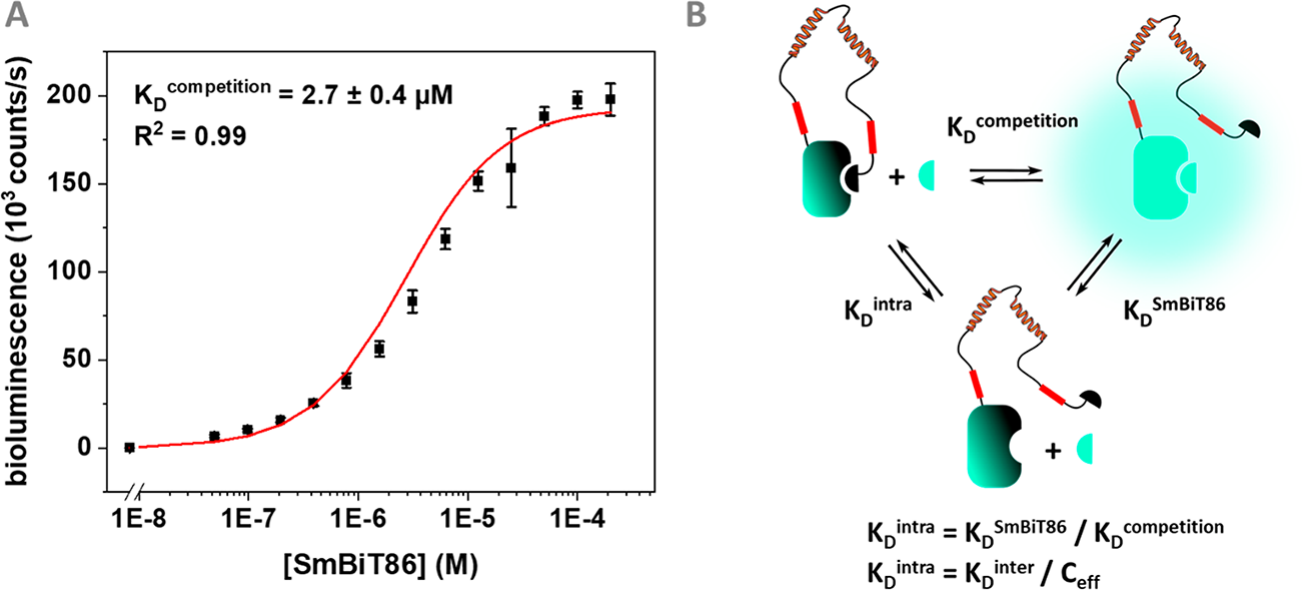
Determining *K*_D_^intra^. (A) Titration to determine *K*_D_^competition^. Experimental conditions: 120 pM LgBiT-DarkBiT101 fusion protein in 1x PBS, 0.02% Tween20, incubation at 4 °C for 16 h. (B) Equilibrium scheme for the autoinhibited LgBiT component based on ref (22) including the equations to calculate *K*_D_^intra^ and subsequently *C*_eff_ for the intramolecular DarkBiT system. Error bars correspond to the standard deviation based on 3 technical replicates.

The efficacy of the DarkBiT peptide to inhibit LgBiT not only depends on its intrinsic affinity (as expressed by the *K*_D_ for intermolecular binding), but also on the effective concentration when both are connected via a linker. We therefore made a fusion protein in which LgBiT was fused to DarkBiT101 using the antibody-responsive linker that was also used in previous anti-HIV1-p17 LUMABS sensors.^12,23^ The blocking efficiency was studied by monitoring bioluminescence activity at increasing concentrations of the (high affinity) SmBiT86 peptide. This titration experiment yielded an apparent *K*_D_^competition^ of 2.7 μM for binding of SmBiT86 to the intramolecularly blocked LgBiT-DarkBiT101 fusion protein (Figure 3A). Based on this value and the *K*_D_ value for binding of SmBiT86 to unblocked LgBiT, *K*_D_^intra^ can be calculated according to the thermodynamic cycle of this system (Figure 3B).^22^*K*_D_^intra^ was found to be 0.0015, meaning that, in the absence of target antibody, DarkBiT101 is bound to LgBiT in 99.85% of the fusion protein population. Since *K*_D_^intra^ = *K*_D_^inter^ /*C*_eff_, this corresponds to a *C*_eff_ of 1.73 mM, which is consistent with model predictions for long flexible linkers bridging a relatively small distance.^24,25^

To investigate which step in the complementation process is rate-limiting, we recorded the increase in bioluminescent signal over time for various SmBiT86 concentrations. The kinetic traces were fitted to a single exponential function to obtain observed rate constants (*k*_obs_) (Figure S2). Interestingly, *k*_obs_ was found to be independent of SmBiT86 concentration (*k*_obs_ ∼ 0.055 min^–1^), which strongly suggests that the DarkBiT101 dissociation is the rate limiting step in the complex formation.

**Figure 4:**
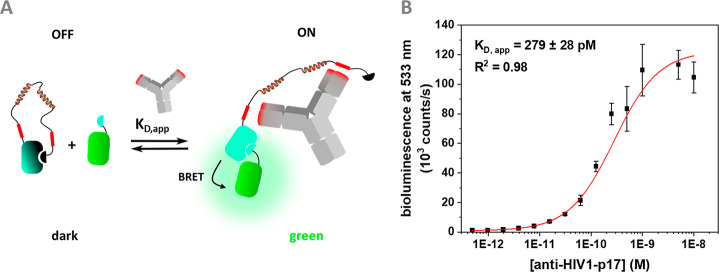
Two component, BRET-based model system. (A) Schematic for the two component system with DarkBiT101 competing with SmBiT99 fused to mNeongreen. (B) Binding experiment for detection of anti-HIV1-p17 using the two component, BRET-based system. Experimental conditions: 50 pM sensor, 50 nM mNeongreen-SmBiT99 in 1×PBS + 1 mg/mL BSA, incubation at 4 °C for 16 h before addition of 1:1000 NanoGlo. Error bars correspond to the standard deviation based on 3 technical replicates.

Having established nearly complete intramolecular complex formation in the absence of target antibody, we next tested whether the DarkBiT101-LgBiT interaction could still be disrupted by antibody binding. To do so, we added 50 nM SmBiT99 (reported *K*_D_ = 180 nM^20,21^) fused to the green fluorescent protein mNeongreen, which upon complexation with nonblocked LgBiT would result in an increase in green luminescence (Figure 4A). Assay conditions were chosen such that the SmBiT99 would not effectively compete with intramolecular binding of DarkBiT101 in the absence of antibody, while still allowing efficient complex formation upon disruption of the DarkBiT101-LgBiT interaction. A titration experiment with anti-HIV1-p17 antibody for this two component sensor system showed a very large, 100 ± 10-fold increase in green bioluminescence upon antibody addition (Figure 4B) and yielded an apparent *K*_D_ of 279 ± 28 pM. This *K*_D_ is similar to that obtained for the original HIV-LUMABS BRET sensor and ∼10-fold higher than that of NB-LUMABS sensor, which might reflect a slightly higher thermodynamic barrier for switch activation. Nonetheless, because the 100-fold increase in bioluminescence intensity renders the system very sensitive, a low LOD of 2 pM was obtained.

### Development of a Single Component dark-LUMABS Sensor

**Figure 5:**
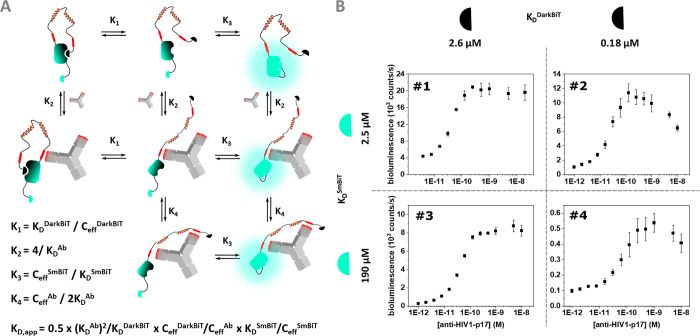
(A) Overall reaction scheme and equilibrium model of dark-LUMABS sensors. The composition of the distinct association constants are listed. The overall *K*_D,app_ of a sensor is the product of the dissociation constants *K*_1_’, *K*_2_’, *K*_3_’, and *K*_4_’ (for details, see the Supporting Information). (B) Antibody titrations for the four tested HIV-NB-LUMABS sensors comprising different SmBiT and DarkBiT affinities. Experimental conditions: Final sensor concentration 20 pM, 1×PBS + 1 mg/mL BSA, final NanoGlo dilution of 1:1000, 16 h incubation at 4 °C. Error bars correspond to the standard deviation of *n* = 3 technical replicates.

While the two-component system shown in Figure 4 already yields an attractive antibody assay, its dependence on two components makes it less robust as its performance will depend on the sensor concentration and be more sensitive to (variations in) matrix conditions. We therefore next constructed a single sensor protein, named dark-LUMABS, that comprises both a (catalytically active) SmBiT domain and a (catalytically inactive) DarkBiT domain that compete for binding to the LgBiT domain (Figure 1D). A thermodynamic equilibrium model was used to guide the design of the dark-LUMABS sensor (Figure 5A). The model predicts that the equilibrium and thus the switch performance for a given antibody concentration is dependent on the intramolecular affinities *K*_D_^DarkBiT^ and *K*_D_^SmBiT^, the affinity of the epitope-antibody binding *K*_D_^Ab^, and the effective concentrations of the intramolecular components (*C*_eff_^Ab^, *C*_eff_^DarkBiT^, *C*_eff_^SmBiT^). The challenge in the design is to tune the relative affinities of the SmBiT and DarkBiT such that luminescence activity is still efficiently suppressed in the absence of antibody, while antibody binding strongly favors formation of the active SmBiT-LgBiT intramolecular complex. We expected that the DarkBiT affinity needed to be higher than that of SmBiT^12^ and tested 4 different sensor variants combining SmBiT affinities of 2.5 and 190 μM with DarkBiT affinities of 2.6 and 0.18 μM.

**Figure 6:**
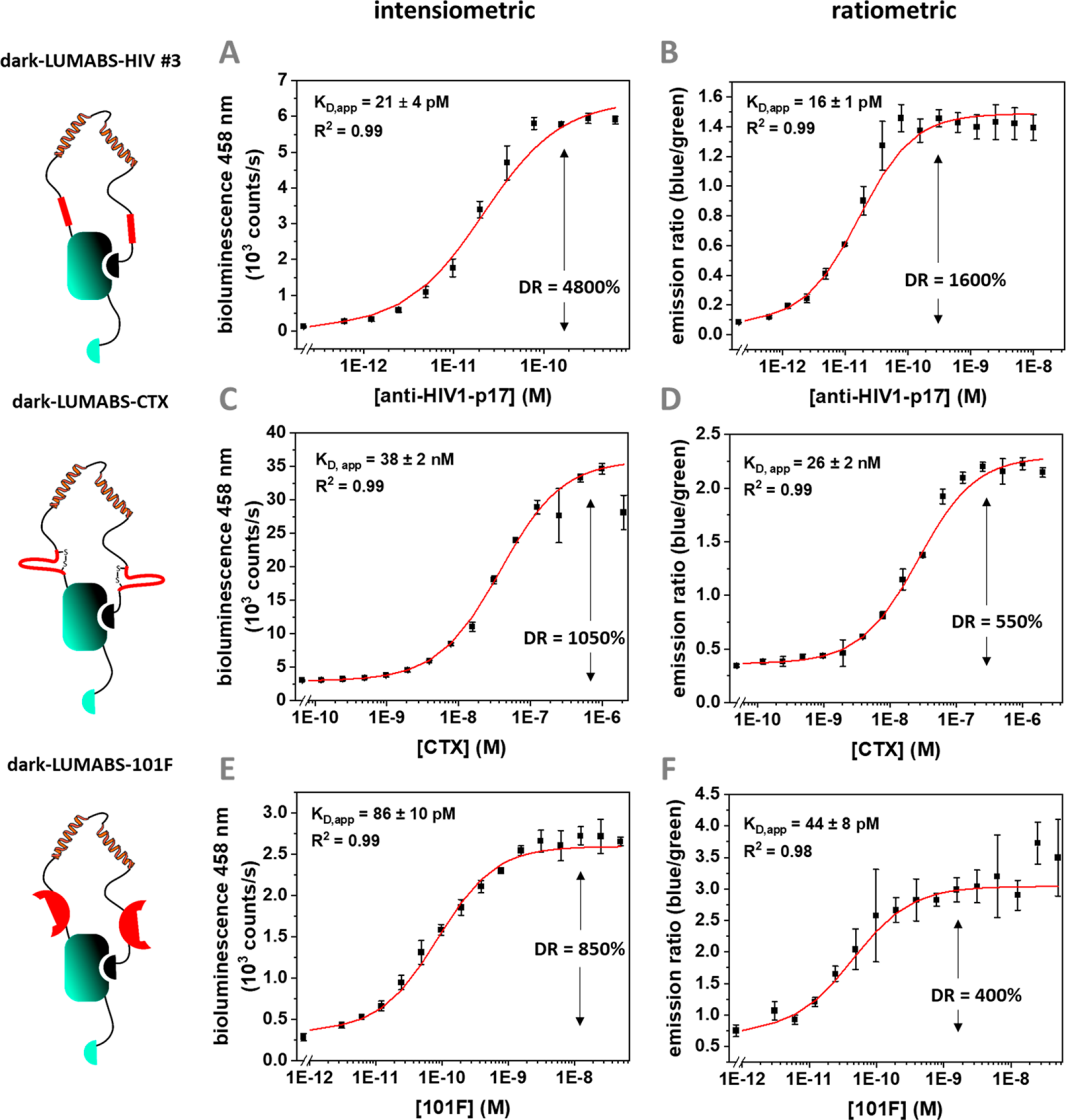
Intensiometric (A,C,E) and ratiometric (B,D,F) binding experiments for dark-LUMABS/target antibody interaction. All experiments were performed in 1×PBS + 1 mg/mL BSA and a final NanoGlo dilution of 1:1000. Binding experiments used low sensor concentrations and were allowed to equilibrate at 4 °C for 16 h to ensure full complex formation even at very low analyte concentration (Figure S7). Ratiometric experiments included low amounts (0.5–1 pM) of calibrator luciferase, allowing to plot the emission ratio between blue (458 nm) and green (533 nm) signal. (A) 20 pM dark-LUMABS-HIV #3; (B) As in (A) but with 0.5 pM calibrator luciferase; (C) 200 pM dark-LUMABS-CTX; (D) as in (C) but with 1 pM calibrator luciferase; (E) 50 pM dark-LUMABS-101F; (F) as in (E) but with 0.5 pM calibrator luciferase. Error bars represent the standard deviation based on 3 technical replicates.

All 4 variants were successfully expressed in *E. coli* and purified to homogeneity using 6xHis and StrepTag II affinity tags. In all cases, the introduction of the DarkBiT resulted in the intended inhibition of split luciferase activity in the OFF-state and a substantial increase in luminescence intensity upon titration with anti-HIV1-p17 antibody. However, consistent differences in sensor properties were observed for the 4 variants (Figure 5B). Using a SmBiT variant with a relatively high affinity (*K*_D_ = 2.5 μM) (#1 and #2) resulted in uncomplete inhibition in the OFF-state, which suggests that the SmBiT already competes with DarkBiT for binding to LgBit in the absence of antibody. Indeed, attenuation of the SmBiT affinity to *K*_D_ = 190 μM effectively restores the low background signal. However, combining the low affinity SmBiT with a high affinity DarkBiT (0.18 μM) suppressed activation of the switch in the presence of antibody, suggesting that this switch (#4) is only partially activated. The optimal sensor performance was observed when combining a SmBiT with *K*_D_ = 190 μM affinity (SmBiT114) with a DarkBiT101, which has a *K*_D_ = 2.6 μM. This optimized sensor dark-LUMABS-HIV #3 showed a 48 ± 8-fold change from complete OFF to complete ON state and an apparent dissociation constant (*K*_D,app_) of 21 ± 4 pM (Figure 6A, Table S1). The absolute luminescence intensity of the fully complemented sensor is 4.4 times lower compared to full-length NanoLuc (Figure S4). Nonetheless, the combination of high sensitivity and the high affinity for antibody binding in this sensor results in a very low limit of detection (LOD) of 0.6 pM, which represents a substantial improvement compared to the previously reported NB-LUMABS^12^ and mNeonG-LUMABS^25^ sensor designs (Table 1, Figure S3).

**Table 1:** Comparison of Relevant NanoLuc-Based Antibody Sensors Including the Ones Developed in This Study

sensor	DR (%)	*K*_D,app_	LOD (pM)	ref
HIV-LUMABS-1	189 ± 2^a^	83 ± 10 pM^a^	10	(10), (26)
Clover4-LUMABS-HIV	808 ± 10^a^	105.7 pM^a^	2.5	(26)
HIV-NB-LUMABS-7	493 ± 13^a^	11.8 ± 0.5 pM^a^	0.6	(12)
**dark-LUMABS-**HIV #3	4818 ± 809^b^	21 ± 4 pM^b^	0.6	this study
	1621 ± 70^a^	16 ± 1 pM^a^		
CTX-LUMABS-2	60 ± 1^a^	55 ± 3 nM^a^	N.D.^c^	(11)
CTX-NB-LUMABS-1	233 ± 12^a^	34.7 ± 3.7 nM^a^	3000	(12)
**dark-LUMABS-CTX**	1046 ± 52^b^	38 ± 2 nM^b^	1000	this study
	550 ± 27^a^	26 ± 2 nM^a^		
3E2H.37-LUMABS	51 ± 3^a^	970 ± 10 pM^a^	167	(4)
**dark-LUMABS-101F**	857 ± 141^b^	86 ± 10 pM^b^	7	this study
	396 ± 67^a^	44 ± 8 pM^a^		

a Ratiometric.

b Intensiometric.

c N.D. not determined.

Sensors with a high affinity DarkBiT domain (#2 and #4; *K*_D_ = 0.18 μM) also showed a decrease in bioluminescence at high antibody concentrations, an effect that is reminiscent of the “hook” effect^27,28^ (Figure 5B). A possible explanation for this phenomenon is that at high antibody concentrations a single sensor protein binds two antibodies, in which case there is no driving force for disrupting the interaction between LgBiT and DarkBiT. We expanded the thermodynamic equilibrium model to also include complexes where two antibodies are bound to a single sensor (Figure S5). Simulations using this model indeed show the observed suppression at higher antibody concentrations, and recapitulate our experimental observation that this effect is most prominent when the affinity of the DarkBiT is high. This result shows the relative affinities of the SmBiT and DarkBiT domains not only determine the switching behavior of the sensor, but also affect the propensity of sensors to form higher order antibody complexes (Figure S6).

### Ratiometric Detection and Extension to Other Antibody Targets

Tuning the relative affinities of the competing DarkBiT and SmBiT components yielded an optimized sensor for anti-HIV1-p17 with a 48 ± 8-fold increase in luminescent intensity. This dynamic range is 10-fold better than the previously reported sensor based on NanoBiT (NB-LUMABS) and 6-fold better than the most optimized BRET-based LUMABS sensor (Clover4-LUMABS-HIV).^26^ This comparison is not completely fair, as the latter represent ratiometric sensor systems, whereas dark-LUMABS-HIV #3 by itself is an intensiometric sensor. However, we recently showed that intensiometric assays can be rendered ratiometric by the addition of green enhanced Nanolantern (GeNL), a tight fusion protein between NanoLuc and mNeongreen.^17,27^ This green-light-emitting NanoLuc variant serves as a calibrator luciferase to correct for changes in luminescent intensities due to substrate turnover and other factors that affect enzyme activity.^28^ Titration experiments in the presence of this calibrator luciferase and ratiometric detection yielded a similar *K*_D_ of 16 ± 1 pM (Figure 6B). The dynamic range was 3-fold lower compared to the one obtained using intensiometric detection (Figure 6A), however, but still improved on the best BRET-based sensor for anti-HIV1-p17 developed so far (Table 1).

To test the general applicability of this design strategy we developed dark-LUMABS variants of two other previously developed antibody sensor proteins targeting the therapeutic anti-EGFR-binding antibody Cetuximab (CTX) and the 101F antibody, which binds to a neutralizing, discontinuous epitope of the F-protein of the respiratory syncytial virus (RSV).^29−31^ Unlike the anti-HIV1-p17 sensor proteins that contain a linear peptide epitope, these LUMABS sensors contain structurally more complex binding domains. The cetuximab sensor contains two disulfide-linked cyclic meditopes that each bind cetuximab with a *K*_D_ of 63 nM. The original LUMABS sensor showed a modest change in emission ratio of 60% that was improved to 233% in the NB-LUMABS format. However, construction of CTX-NB-LUMABS required introduction of the non-natural amino acid pAzF to enable site-specific coupling of a Cy3 label using Strain-Promoted Azide–Alkyne Cycloaddition (SPAAC). Cy3 acted as BRET acceptor in the OFF state of the switch and allowed ratiometric detection.^12^ Dark-LUMABS-CTX was obtained by simply replacing the anti-HIV1-p17 epitopes in dark-LUMABS-HIV#3 with the cyclic peptide meditopes binding to CTX. Without any further optimization, dark-LUMABS-CTX showed a strongly improved dynamic range of 1050% and 550% for intensiometric and ratiometric detection, respectively (Table 1, Figure 6C and D).

The 101F sensor contains two *de novo* designed domains that mimic a discontinuous epitope and bind the 101F antibody with a monovalent *K*_D_ of 20 nM.^4^ The original 101F LUMABS sensor could detect high pM levels of 101F, but showed a modest change in emission ratio of 51 ± 3%. Introduction of these *de novo* binding domains in the dark-LUMABS format yielded the dark-LUMABS-101F sensor, which displayed an improved dynamic range of 850% and 400% for intensiometric and ratiometric detection, respectively (Table 1, Figure 6E and F), representing a 10-fold increase in sensitivity.

### Characterization of Sensor Switching Kinetics

All titration experiments reported sofar were done using an incubation time of 16 h at 4 °C to ensure full equilibration, and thus reliable determination of *K*_D,app_ values, also at low pM antibody concentrations (Figure S7). However, the performance of a sensor is not only determined by thermodynamic parameters such as affinity and sensitivity, but also the kinetics of the sensor response. The use of the calibrator luciferase allowed us to also monitor the sensor response in time as a function of antibody concentration at 22 °C (Figure S8). Considering that DarkBiT101 dissociation was found to be the rate limiting step in binding of the SmBit86 peptide to LgBiT-DarkBiT101 fusion protein (Figures 3B and S2), we expected that activation of the dark-LUMABS sensors would also be independent of antibody concentration and display similar kinetics. Surprisingly, the observed rate constants were found to be clearly dependent on antibody concentration for both the dark-LUMABS-HIV #3 and dark-LUMABS-CTX sensors, reaching full activation within 20 min at high antibody concentration (Figure S8). In contrast, the kinetics of dark-LUMABS-101F activation was found to be independent of antibody concentration, but the observed rate constant for the latter sensor was about 5-fold faster than that of the LgBiT-DarkBiT101 switch. Interestingly, these results suggest that antibody binding can affect the dissociation of the DarkBiT101 in the sensor construct. At this point, the mechanism of this effect remains elusive, but one possibility is that at least in some antibody sensors bivalent binding of the antibody to the sensor promotes the dissociation of the DarkBiT101 peptide. We also compared the kinetic performance of the dark-LUMABS-HIV #3 and the dark-LUMABS-101F sensors directly with the previously developed NB-LUMABS-HIV and 3E2H.37-LUMABS sensors, respectively (Figures S9 and 10). With optimized dark-LUMABS sensor and calibrator concentration, target concentrations of 10 pM (anti-HIV1-p17) and 100 pM (101F) were detectable against background within 30 min at 22 °C, which is better (anti-HIV1-p17) or similar (101F) compared to the previous versions of the LUMABS sensors (Figures S9 and 10).

## Conclusion and Outlook

In this work, we introduced the use of catalytically inactive SmBiT variants (DarkBiTs) as an attractive, generally applicable strategy to suppress background signal, and hence increase sensitivity, in bioluminescent sensors based on split NanoLuc complementation. First we established that exchanging the catalytically important arginine in SmBiT to alanine in DarkBiT does not change the binding affinity to LgBiT, which is surprising given the significant difference in charge and size between these amino acids. This finding is important, because it means that DarkBiTs can be generated with the same range of affinities from low nanomolar to high miromolar as their corresponding SmBiT variants.^20^ Fusing a DarkBiT domain with *a* a *K*_D_ of 2.6 μM to LgBiT via an antibody-responsive semiflexible linker resulted in nearly complete (99.85%) inhibition of LgBiT. Using the anti-HIV1-p17 antibody as a first target antibody, systematic tuning of competing SmBiT and DarkBiT affinities yielded a sensor with a remarkable dynamic range of 48 ± 8-fold (4800%) and 16 ± 1-fold (1600%) for intensiometric and ratiometric measurements, respectively. Moreover, the design of the dark-LUMABS system was easily adapted to different antibodies, as demonstrated for antibodies targeting cetuximab and a discontinuous epitope of the respiratory syncytial virus (RSV). In all three cases the dark-LUMABS sensor format outperformed both the original LUMABS and the NB-LUMABS sensor formats. The affinities of the new sensors were at least similar to those of their parent LUMABS sensors (HIV and CTX) or even further improved (101F) (Table 1, Figure 6).

The dark-LUMABS system is highly modular, as simple replacement of the binding domains of the optimized dark-LUMABS-HIV#3 sensor yields dark-LUMABS sensor variants with similar dynamic ranges even without additional optimization. The modularity of the dark-LUMABS platform allows one to predict and optimize the sensor properties by tuning the affinities of the SmBiT and LgBiT components. The equilibrium scheme and the expanded thermodynamic model show that the sensor properties are not only dependent on the SmBiT and DarkBiT affinities, but also on the strength of the antibody–antigen interaction and three effective concentration terms, *C*_eff_^DarkBiT^, *C*_eff_^SmBiT^, and *C*_eff_^Ab^ (Figure S11). *C*_eff_^DarkBiT^ was experimentally determined as 1.73 mM, while *C*_eff_^SmBiT^ can be estimated to be 4.7 mM based on previously developed models for GGS linkers,^25^ leaving *C*_eff_^Ab^ as the only unknown parameter. Assuming an affinity of 42 nM for the monovalent antibody-peptide epitope interaction used in LUMABS-HIV#3 and the experimentally determined K_D,app_ would yield a *C*_eff_^Ab^ of 1.1 mM. Please note that the value of *C*_eff_^Ab^ may be different for different antibodies, as the effective distance that the semiflexible linker needs to bridge may be different depending on the architecture of the antibody-sensor complex. Nonetheless, the model provides a useful starting point to guide the design of dark-LUMABS sensors for other antibody targets.

The use of autoinhibited split-NanoLuc systems introduced here is likely to be more broadly applicable, both to improve the performance of existing sensors and the construction of sensors for novel targets, provided they can be designed to undergo a sufficiently large conformational change to disrupt the LgBiT-DarkBiT interaction. For instance, intensiometric sensors such as NanoLuc-based protease sensors^32^ or NanoBiT-based genetically encoded Ca^2+^ sensors^33^ could be improved by reducing the bioluminescent background signal in the OFF-state, resulting in higher dynamic ranges. Intramolecular inhibition of LgBiT activation could also further improve the performance of sensors based on intermolecular complexation, providing an alternative caging strategy for the one used in the lucCage system that requires sophisticated protein engineering. Furthermore, three-state sensors (dark, BRET-OFF, BRET-ON) could be envisioned by combining LgBiT, DarkBiT, SmBiT, and SmBiT with the BRET acceptor with possible applications in multistate molecular tension sensors^34^ or nucleic acid detection.^35^ Our work thus establishes intramolecular inhibition as a powerful concept to control biomolecular switches, both in natural systems and in the engineering of new protein sensors and switches.^36−38^

## Supporting Information Available

Materials and methods; information about Python simulations; properties of dark-LUMABS-HIV variants; unspecific binding of SmBiT peptides to BSA; kinetics of SmBiT86 peptide binding; overview of LOD determination; comparison of full-length NanoLuc and NanoBiT; expanded thermodynamic equilibrium model; simulations of *K*_D_^SmBiT^/*K*_D_^DarkBiT^ changes; comparison of incubation times; kinetic measurements and *k*_obs_ plots; kinetic behavior of anti-HIV1-p17 sensors; kinetic behavior of 101F sensors; simulations of *K*_D_^Ab^/*C*_eff_^Ab^ changes (PDF)
