# Supplementary material for: Bioluminescence Goes Dark: Boosting the Performance of Bioluminescent Sensor Proteins Using Complementation Inhibitors
Source: ACS Sens. 2022 Nov 30;7(12):3800–8. doi: 10.1021/acssensors.2c01726 (PMC9791688; doi:10.1021/acssensors.2c01726)
Supplement: Supplementary file 1 — se2c01726_si_001.pdf [file se2c01726_si_001.pdf]

Supporting information

Bioluminescence goes dark: boosting the performance of bioluminescent sensor proteins using complementation inhibitors

Alexander Gräwe, Maarten Merkx

Laboratory of Protein Engineering, Department of Biomedical Engineering, Eindhoven University of Technology, Eindhoven, the Netherlands; Institute for Complex Molecular Systems, Eindhoven University of Technology, Eindhoven, the Netherlands.

Table of contents

Supplementary Materials and Methods S2
- Materials S2
- Cloning S2
- Protein purification S2
- Thermodynamic equilibrium scheme S2
- Calculation of effective concentrations S3
- Thermodynamic equilibrium simulations S3
- Titrations S4
- DarkBiT101 affinity S4
- Fitting of single exponential binding processes S5

Supplementary Tables and Figures S6
- Table S1: Properties of dark-LUMABS-HIV variants S6
- Figure S1: Unspecific binding of SmBiT peptides to BSA S7
- Figure S2: Kinetics of SmBiT86 peptide binding S7
- Figure S3: Overview of Limit of Detection (LoD) determination S8
- Figure S4: Comparison of full-length NanoLuc and NanoBiT S9
- Figure S5: Expanded thermodynamic equilibrium model S9
- Figure S6: Simulations of K_D^{SmBiT} / $K_D^{DarkBiT}$ changes S10
- Figure S7: Comparison of incubation times S10
- Figure S8: Kinetic measurements and k_{obs} plots S11
- Figure S9: Kinetic behavior of anti-HIV1-p17 sensors S12
- Figure S10: Kinetic behavior of 101F sensors S13
- Figure S11: Simulations of K_D^{Ab} / C_{eff}^{Ab} changes S13

Peptide & Protein sequences S14

References S17

Supplementary Materials and Methods

Materials

Peptides with acetylated N-terminus and amidated C-terminus were purchased from GenScript (SB86: VSGWRLFKKIS, ≥75% purity; DarkBiT 101: SVTGYALFEKESG, ≥75% purity). Terminal S and G were added to the DarkBiT peptide to account for linker amino acids in fusion proteins. NanoGlo substrate was purchased from Promega. Unless stated otherwise, chemicals were purchased from Sigma.

Cloning

DNA oligonucleotides were purchased from IDT. To obtain dark-LUMABS variants, existing pET28a(+) NB-LUMABS were mutated via Ligase Cycling reaction (*1, 2*) or annealed oligo cloning using Spe1 and Bsa1 restriction sites present in the NB-LUMABS plasmids. Successful cloning was confirmed via Sanger Sequencing (BaseClear).

Protein purification

For purification of dark-LUMABS variants and calibrator luciferase, *E. coli* BL21 (DE3) cells were transformed with corresponding pET28a(+) plasmids. Cells were grown in LB medium supplemented with 50 µg/ml kanamycin at 37 °C at 180 rpm in a shaking incubator. Large cultures (750 ml) in 2 l baffled flasks were inoculated with corresponding overnight cultures and induced with IPTG at OD_{600} 0.6-0.8. Proteins were expressed overnight at 18 °C. Harvested cells were lysed with BugBuster reagent (Novagen) supplemented with Benzonase (Merck), and proteins were purified with Ni-NTA chromatography followed by Strep-Tactin XT (iba) using gravity flow columns. Protein purity was confirmed by reducing SDS-PAGE and concentrations calculated using A_{280} nanodrop measurements and the corresponding extinction coefficients (based on protein sequence). Proteins in Strep-Tactin XT elution buffer (150 mM NaCl, 100 mM Tris-Cl pH 8, 1 mM EDTA, 50 mM D-biotin) were aliquoted, flash frozen in liquid N_2 and stored at -70 °C. NB-LUMABS-HIV6 was expressed and prepared as described in (*3*), 3E2H.37-LUMABS was expressed and purified as described in (*4*).

Thermodynamic equilibrium scheme

The equilibrium scheme that was devised in the main text Figure 5A is adapted from previous works on the LUMABS and RAPPID platforms (*5, 6*). The equilibrium constants are defined as follows: K_1, intramolecular release of the DarkBiT from LgBiT; K_2, monovalent antibody binding based on the 4 possible binding combinations and the monovalent affinity of the epitope K_D^{Ab}; K_3, intramolecular binding of SmBiT to LgBiT; K_4, formation of the bivalent complex. The equilibrium constants take effective concentrations (C_{eff}) into account that result from intramolecular linkage (for DarkBiT and SmBiT) or intermolecular recruitment (for the bivalent complex formation). The overall apparent dissociation constant $K_{D,app}$ is the product of the inversed equilibrium constants K_1 to K_4 (*5*) and can be written as

$$K_{D,app} = \frac{1}{2} \times \frac{\left(K_D{}^{Ab}\right)^2}{K_D{}^{DarkBiT}} \times \frac{C_{eff}{}^{DarkBiT}}{C_{eff}{}^{Ab}} \times \frac{K_D{}^{SmBiT}}{C_{eff}{}^{SmBiT}}$$

With all other parameters known, C_{eff}^{Ab} for each sensor could be calculated from this equation.

Calculation of effective concentrations

The term effective concentration C_{eff} connects an intramolecular affinity to an intermolecular affinity (*7*) and is defined as

$$C_{eff} = K_D^{inter} / K_D^{intra}$$

$C_{eff}^{DarkBiT}$ was derived from competition experiments as 1.73 mM (see main text). As the SmBiT is connected to the LgBiT with a well-understood, flexible GGS linker, the C_{eff}^{SmBiT} was determined with the effective concentration calculator app (*8*), considering a persistence length of 3.7 Å (*9*) and distance of 30 Å between the N-terminus of LgBiT and the binding site of SmBiT measured using Chimera and the PDB file 5IBO. The app gave a C_{eff}^{SmBiT} of 4.66 mM.

Thermodynamic equilibrium simulations using the expanded model

To understand how the hook effect is affected by changes in the sensors intrinsic properties, simulations were performed with a general python model framework developed by Geertjens *et al.* (*10*) in the Spyder environment. The framework is freely available under DOI: 10.5281/zenodo.5531622. The mass balance equations used to build the model derive from the expanded thermodynamic equilibrium scheme shown in Figure S3, and were written in python as:

Sensoro=Sensord; KDDarkBiT/MeffDarkBiT

Sensoro=Sensora; KDSmBiT/MeffSmBiT

Sensoro+Ab1=Ab1Sensoro; Kab/4

Sensord+Ab1=Ab1Sensord; Kab/4

Sensora+Ab1=Ab1Sensora; Kab/4

Ab1Sensoro+Ab1=Ab1Ab1Sensoro; Kab/2

Ab1Sensora+Ab1=Ab1Ab1Sensora; Kab/2

Ab1Sensord+Ab1=Ab1Ab1Sensord; Kab/2

Ab1Sensoro=Ab1Sensoroo; (2*Kab)/Meffab

Ab1Sensora=Ab1Sensoraa; (2*Kab)/Meffab

Labeled specie:

data_mode: custom

custom input: (Ab1Sensoraa+Ab1Ab1Sensora+Ab1Sensora+Sensora)*constant

o/a/d suffixes relate to LgBiT in an open state, active state (bound to SmBiT) or dark state (bound to DarkBiT). The hook effect is represented by the equations in green font color. To simulate changes, all involved terms (K_Ds, C_{eff}s) were kept constant except the term of interest.

Titrations

Unless specified otherwise, all titrations were performed in 1xPBS (10 mM phosphate buffer, 2.7 mM KCl and 137 mM NaCl, pH 7.4) supplemented with 1 mg/ml BSA to avoid unspecific adsorption of proteins to surfaces. As additive, BSA worked better and gave higher signal intensities than Tween20 in all experiments except titrations with SmBiT peptides. Assay components were diluted in the same buffer before measurements. Unless stated otherwise, the sensor proteins were incubated with different concentrations of target antibody for 16 h at 4 °C to allow equilibration, before NanoGlo substrate (furimazine) was added from a freshly prepared 10x stock directly before the measurement to obtain the final volume and concentrations. Unless stated otherwise, the final NanoGlo dilution during the measurements was 1:2000. Binding assays were performed in white 364-well plates (flat bottom, Nunc, ThermoFisher) and a total volume of 20 µl with n=3 technical replicates. Unless stated otherwise, bioluminescence at 458 nm was determined from bioluminescence spectra (398-653 nm, integration time 100 ms) measured at 20 °C in a TECAN Spark 10M. Resulting binding curves were fitted to the standard binding model (P * x) / (K_D + x) + c. Dynamic range (DR) was calculated via

$$\mathrm{DR} = \frac{\mathrm{Max} - \mathrm{Min}}{\mathrm{Min}}$$

With *Max* being the signal at saturated sensor (mean of n=3) and *Min* the signal in absence of the target (mean of n=3). DR uncertainties were propagated from the standard deviation of *Max* and *Min* (*s(Max)* and *s(Min)*, respectively) using (*11*)

$$\frac{1}{Min^2}\sqrt{(Max \cdot s(Min))^2 + (Min \cdot s(Max))^2}$$

DarkBiT101 affinity

For data evaluation of competitive binding based on Motulsky and Neubig (*12*), DarkBiT101 concentrations were converted to logarithmic values and the bioluminescence signals were fitted to

$$y = background + \frac{maximum\ signal - background}{1 + 10^{x - \log IC_{50}}}$$

The background was constrained to positive values. Fitting was performed in Origin 2020. The K_i was then determined from

$$K_i = \frac{IC_{50}}{1 + \frac{[SmBiT86]}{K_D}}$$

with the concentration of active SmBiT86 [SmBiT86] and its K_D for LgBiT binding that was determined in a parallel binding experiment (main text **Figure 2A**).

Fitting of single exponential binding processes

To determine the observed rate constants of the association reactions, k_{obs}, in kinetic experiments, the traces were fit to a single exponential binding process using (*13*)

$$Y = -Y_{eq} \cdot e^{-k_{obs} \cdot t} + Y_{eq}$$

Where Y is the observed blue-to-green ratio, Y_{eq} is the final equilibrium value of the blue-to-green ratio, k_{obs} is the observed rate constant of the respective association reaction, and *t* is the time in minutes.

Supplementary Tables and Figures

Table S1 Properties of dark-LUMABS-HIV variants tested in this study. The titration curves in Figure 5B (main text) were fitted until the start of the hook effect with a 1:1 binding model to obtain $K_{D,app}$ values presented here.

#	Affinity level		$K_{D,app}$	Dynamic Range (fold change)
1	SmBiT	2.5 µM	58 ± 14 pM	4 ± 0
	DarkBiT	2.5 µM		
2	SmBiT	2.5 µM	31 ± 5 pM	10 ± 1
	DarkBiT	0.18 µM		
3	SmBiT	190 µM	21 ± 4 pM	48 ± 8
	DarkBiT	2.5 µM		
4	SmBiT	190 µM	94 ± 21 pM	4 ± 1
	DarkBiT	0.18 µM		

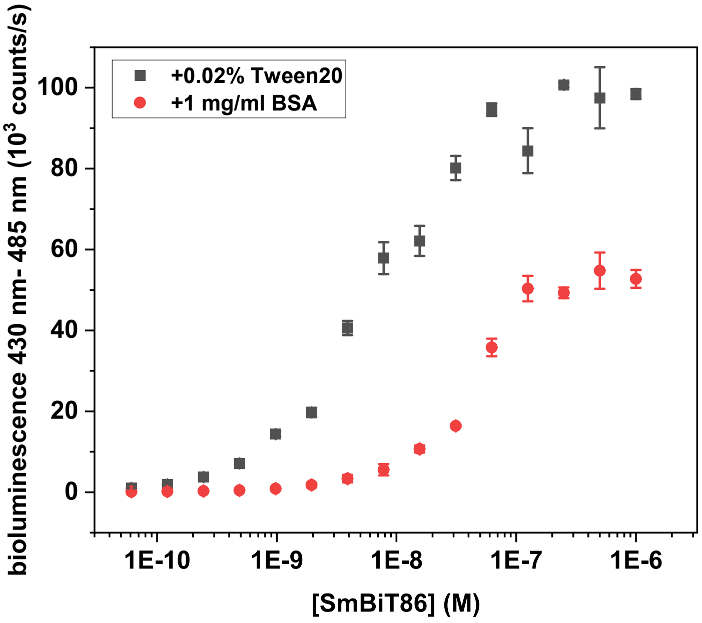

Figure S1 BSA affects the intensity of the luminescent signal and decreases the apparent affinity in titration experiments of SmBiT peptide binding to LgBiT. Further experimental conditions: 1xPBS plus indicated supplement, 15 pM LgBiT, 1:1000 diluted NanoGlo, 1h incubation at room temperature (22 °C).

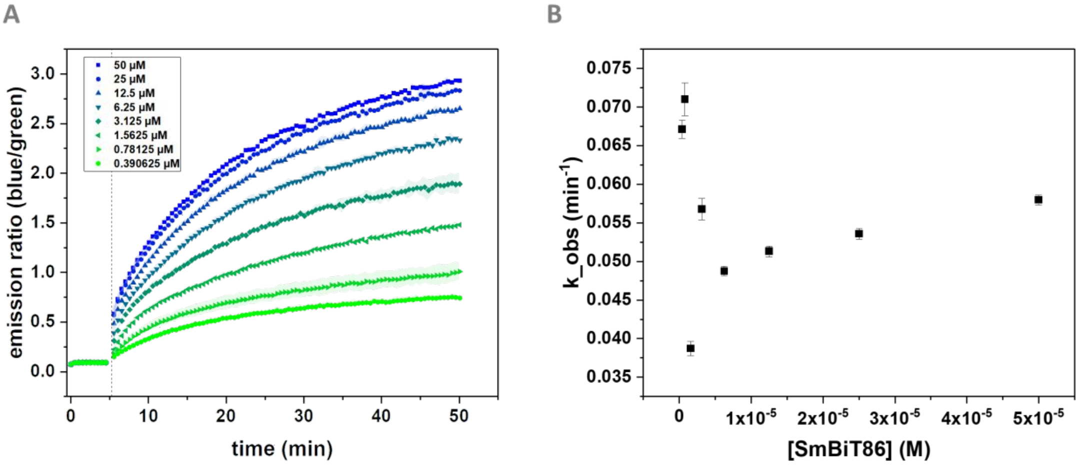

Figure S2 Time-course, ratiometric experiment of the SmBiT86 peptide binding to to the intramolecularly blocked LgBiT-DarkBiT101 fusion protein. A) Legend indicates the final concentrations of SmBiT86, dashed line indicates addition of SmBiT86 (20 µl) to preincubated sensor, calibrator and substrate (20 µl). The kinetic traces after SmBiT86 addition were fitted to $Y = -Y_{eq} *e^{(-k_{obs}*t)}+Y_{eq}$ and k_{obs} values were plotted as a function of [SmBiT86] (B). Further experimental condition: 1xPBS +0.02% Tween20, final concentrations: 120 pM of LgBiT-DarkBiT101, 10 pM calibrator (GeNL), 1:1000 diluted NanoGlo, 22 °C, total volume after SmBiT86 addition: 40 µl.

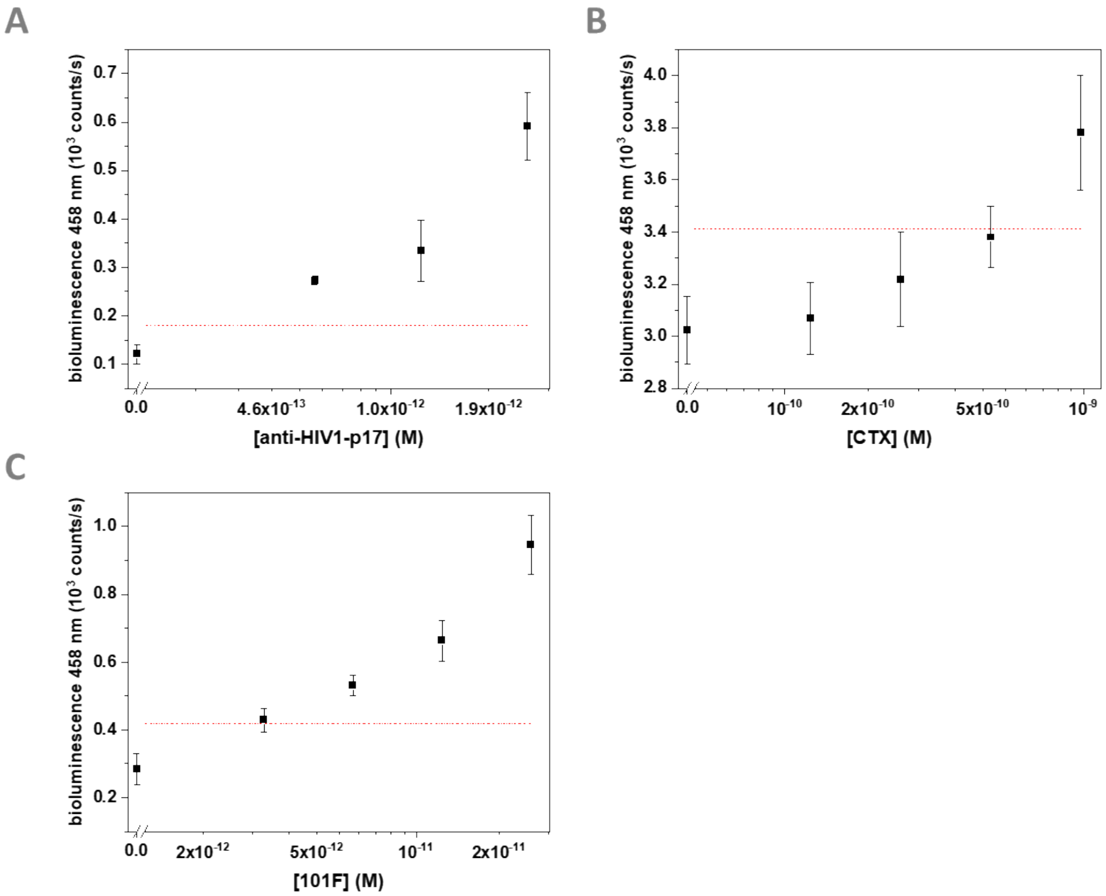

Figure S3 Overview of Limit of Detection (LoD) determination. The shown graphs are zoom-ins of Fig 6 A,C and E in the main text. By applying the 3σ rule (mean of blank plus 3 times its standard deviation), the limit of detection for each antibody sensor was calculated and the corresponding bioluminescent signal is shown as red, dotted line in the figure. The reported LoD is then the first value with an intensity above this line. A) anti-HIV1-p17 dark-LUMABS, B) CTX-dark-LUMABS, C) 101F-dark-LUMABS. The values are listed in table 1 in the main text. The experimental conditions, as mentioned in Figure 6 in the main text, are as follows: A) 20 pM dark-LUMABS-HIV #3, B) 200 pM dark-LUMABS-CTX; C) 50 pM dark-LUMABS-101F. All experiments were performed in 1x PBS +1mg/ml BSA and a final NanoGlo dilution of 1:1000. The sensor proteins were incubated with different concentrations of target antibody for 16 h at 4 °C to allow equilibration, before NanoGlo substrate (furimazine) was added from a freshly prepared 10x stock directly before the measurement to obtain the final volume (20 µl) and concentrations. Error bars represent standard deviation based on 3 technical replicates.

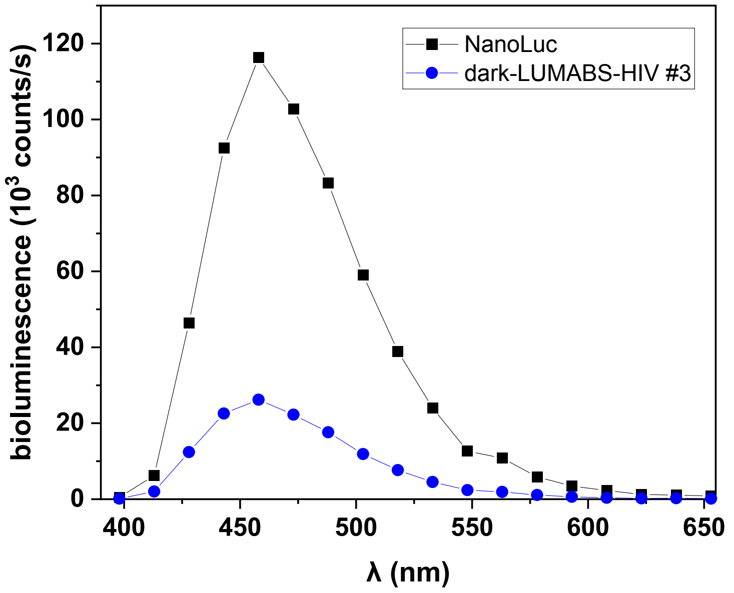

Figure S4 Signal intensity comparison between full-length NanoLuc and dark-LUMABS-HIV #3 in presence of target antibody. The bioluminescent signal at 458 nm of the activated split luciferase (NanoBiT) in the dark-LUMABS switch is 4.4 times lower compared to the signal of full-length NanoLuc at the same protein concentration (200 pM). Experimental conditions: 1x PBS +1mg/ml BSA, 1 nM anti-HIV1-p17, 200 pM full-length NanoLuc or dark-LUMABS-HIV #3, 1:1000 NanoGlo substrate. 1 h incubation time at room temperature (22 °C) before addition of NanoGlo and subsequent measurement at 22 °C.

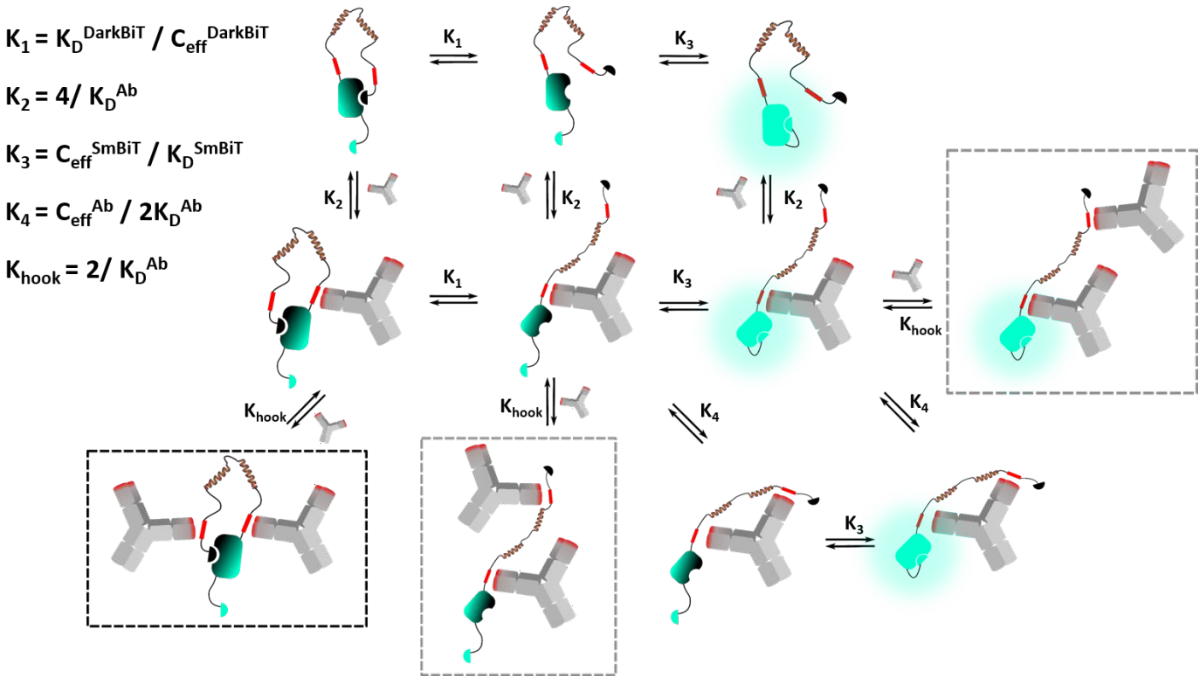

Figure S5 Expanded thermodynamic equilibrium model including the hook effect (species in dashed-line rectangles). The hook effect in black dashed lines has the strongest effect. K_{hook} considers that a second antibody can bind in 2 ways to the sensor (hook effect) unless both epitope regions are already bound to one antibody.

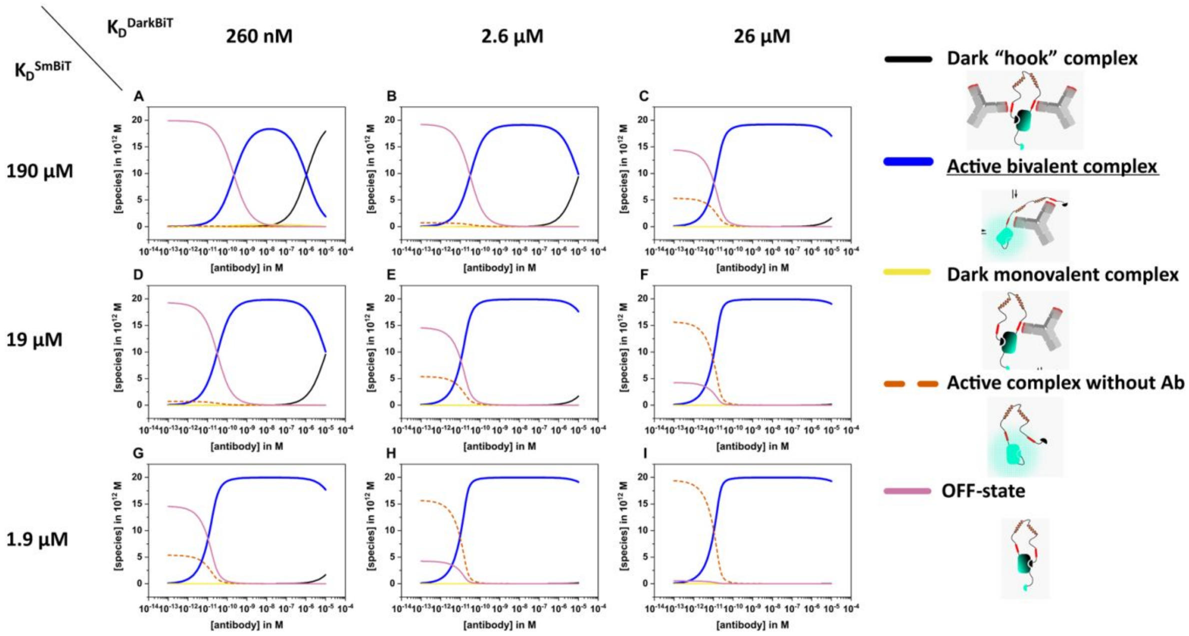

Figure S6 Simulations using the expanded thermodynamic equilibrium model confirm stronger hook effect for higher DarkBiT affinities (lower $K_D^{DarkBiT}$) in Panel A. Sensor concentration was set at 20 pM. All other parameters (in M) were left as follows: $C_{eff}^{DarkBiT}$:0.00173, K_D^{Ab}:4.2E-8, C_{eff}^{SmBiT}:4.66E-3, C_{eff}^{Ab}:1.14E-3.

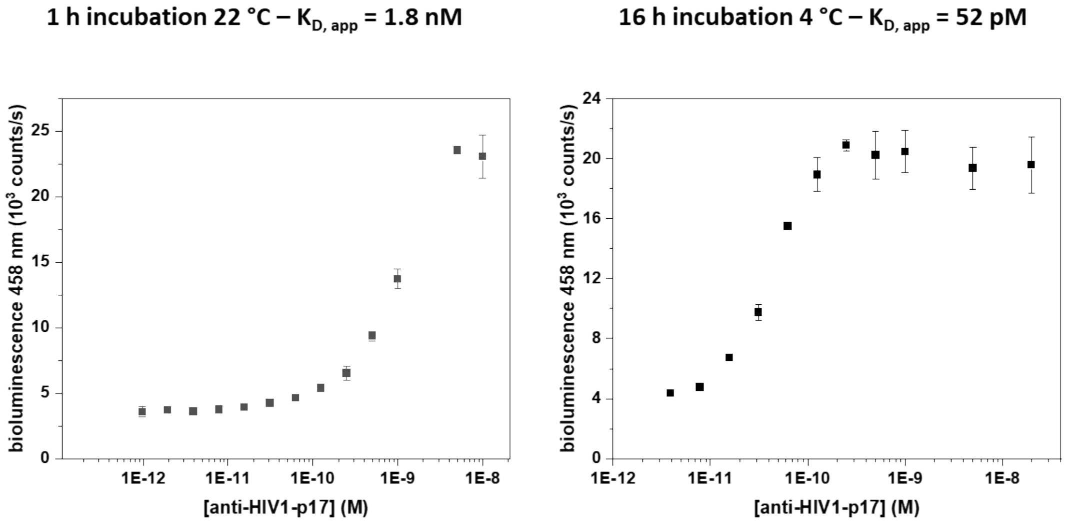

Figure S7 Longer incubation times are necessary to reach binding equilibrium and determine $K_{D,app}$. Experimental conditions: 1xPBS +1mg/ml BSA, 20 pM dark-LUMABS-HIV #1, 1:2000 diluted NanoGlo, temperature and incubation time as written above the graphs.

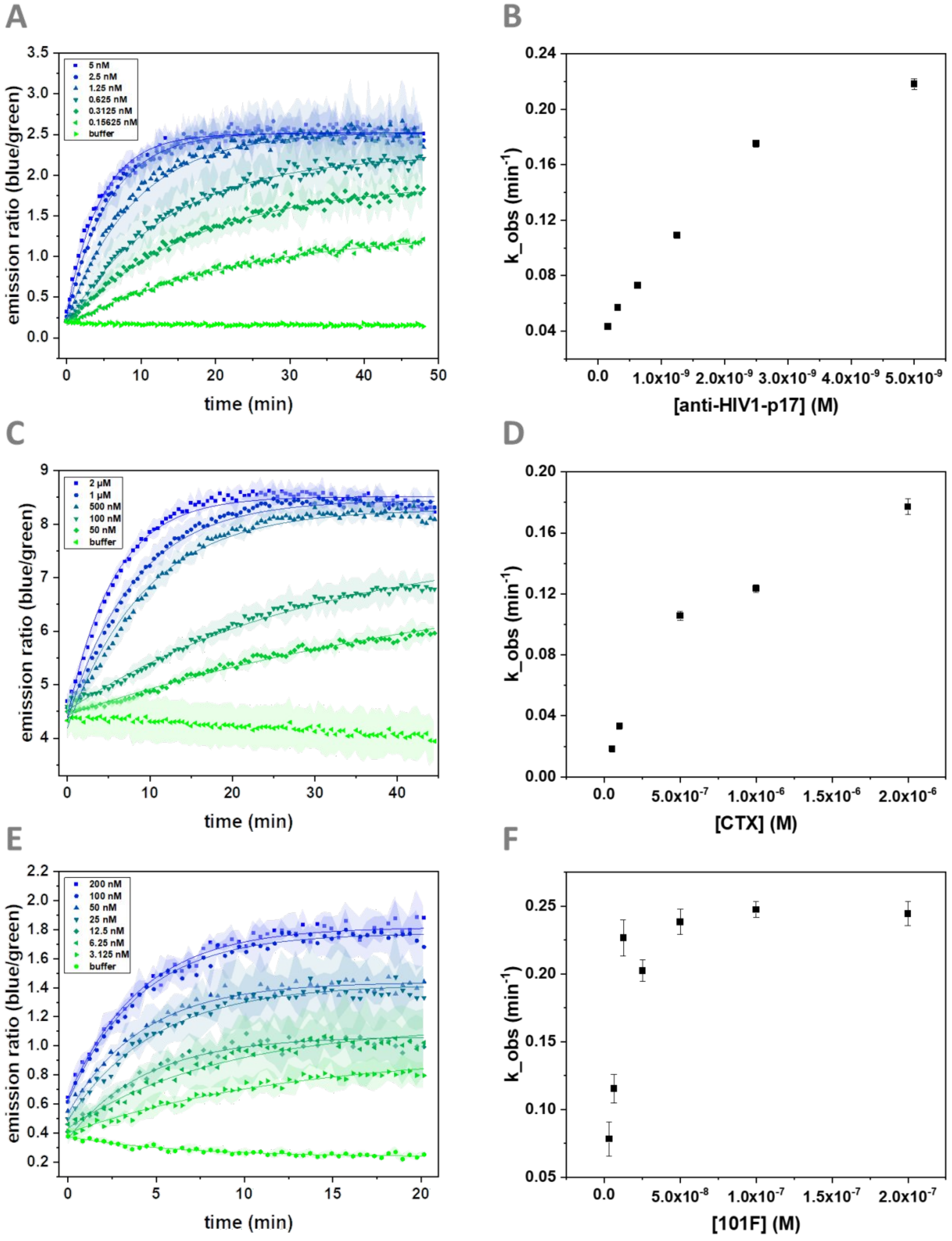

Figure S8 Kinetic measurements and k_{obs} plots of dark-LUMABS-HIV #3 (A&B), dark-LUMABS-CTX (C&D), and dark-LUMABS-101F (E&F) to investigate if the sensors activation is dependent on the target concentration. The time on the x-axis in A,C and E refers to the time after target antibody addition. For the 101F measurement, the fitting was constrained to 21 min of the kinetic traces to achieve fits with $R^2 > 0.95$, as the blue/green ratio started decreasing afterwards. Experimental conditions: 1xPBS + 1mg/ml BSA, 1:1000 NanoGlo substrate, 22 °C. n=3 technical replicates per concentration. A) 0.2 nM of dark-LUMABS-HIV #3 sensor protein, 0.8 pM of calibrator (GeNL); C) 10 nM of dark-LUMABS-CTX sensor protein, 5 pM calibrator (GeNL); E) 1 nM of dark-LUMABS-101F sensor protein, 0.8 pM calibrator (GeNL). The kinetic traces were fitted to a single exponential binding process using $Y = -Y_{eq} * e^{(-k_{obs}*t)} + Y_{eq}$ according to (*13*). The k_{obs} values of the fitting results were plotted as a function of antibody concentrations to check for concentration dependency.

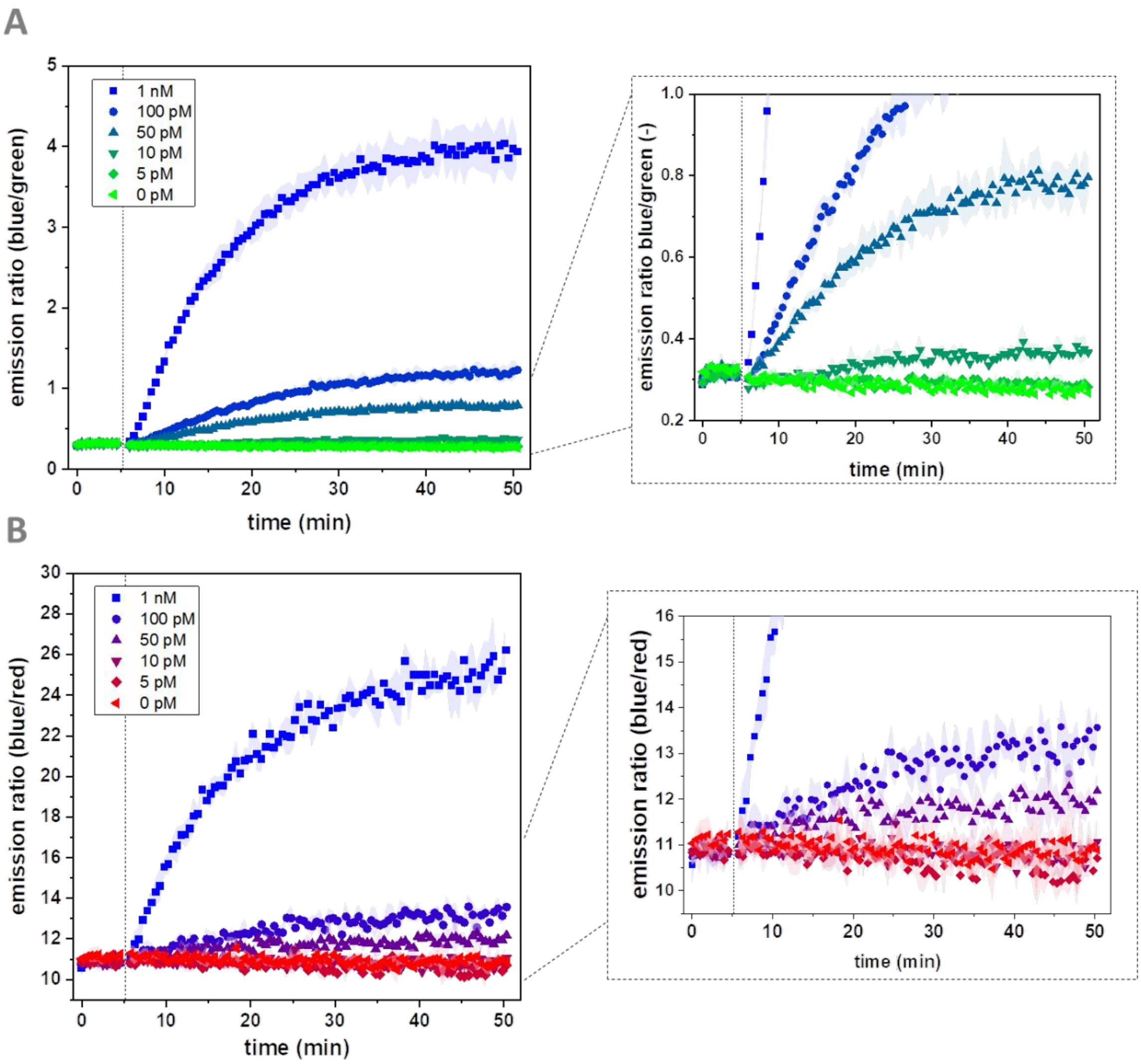

Figure S9 Kinetic behavior of anti-HIV1-p17 sensors. Legends indicates the concentration of anti-HIV1-p17 used, dashed line indicates addition of antibody to preincubated sensor, calibrator (in A) and substrate. A) dark-LUMABS-HIV #3 at 400 pM sensor and 2 pM calibrator luciferase. Zoom-in shows increase in blue/green ratio against background for 10 pM of target, detectable in a microwell platereader. Dynamic range between 0 and 1 nM target (t = 50 min): 1373 %. B) NB-LUMABS-HIV6 at 400 pM sensor. Zoom-in shows increase in blue/red ratio against background for lower target concentrations. Dynamic range between 0 and 1 nM target (t = 50 min): 141 %. Further experimental conditions: 1xPBS +1mg/ml BSA with 1:1000 NanoGlo substrate, 22 °C . n=3 technical replicates per concentration.

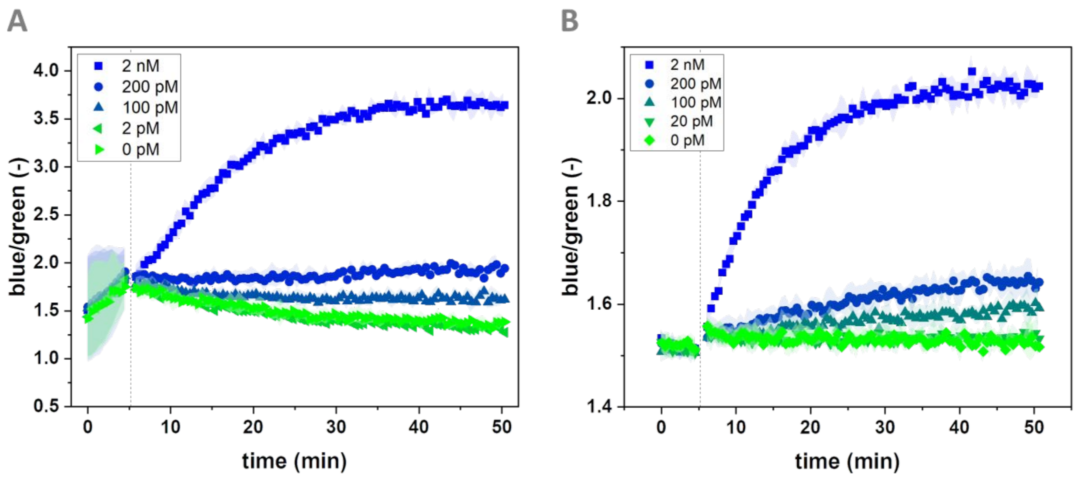

Figure S10 Kinetic behavior of 101F sensors. Legends indicates the concentration of target antibody used, dashed line indicates timepoint of antibody addition. A) dark-LUMABS-101F. 10 nM sensor and 2 pM calibrator were used in a final assay volume of 20 µl. Dynamic range between 0 and 2 nM target at t=50 min: 163%. B) 3E2H.37-LUMABS. Sensor concentration 400 pM, calibrator concentration 1 pM. Dynamic range between 0 and 2 nM target at t=50 min: 25 %. Further experimental conditions: 1xPBS + 1mg/ml BSA, 1:1000 NanoGlo substrate, 22 °C. n=3 technical replicates per concentration.

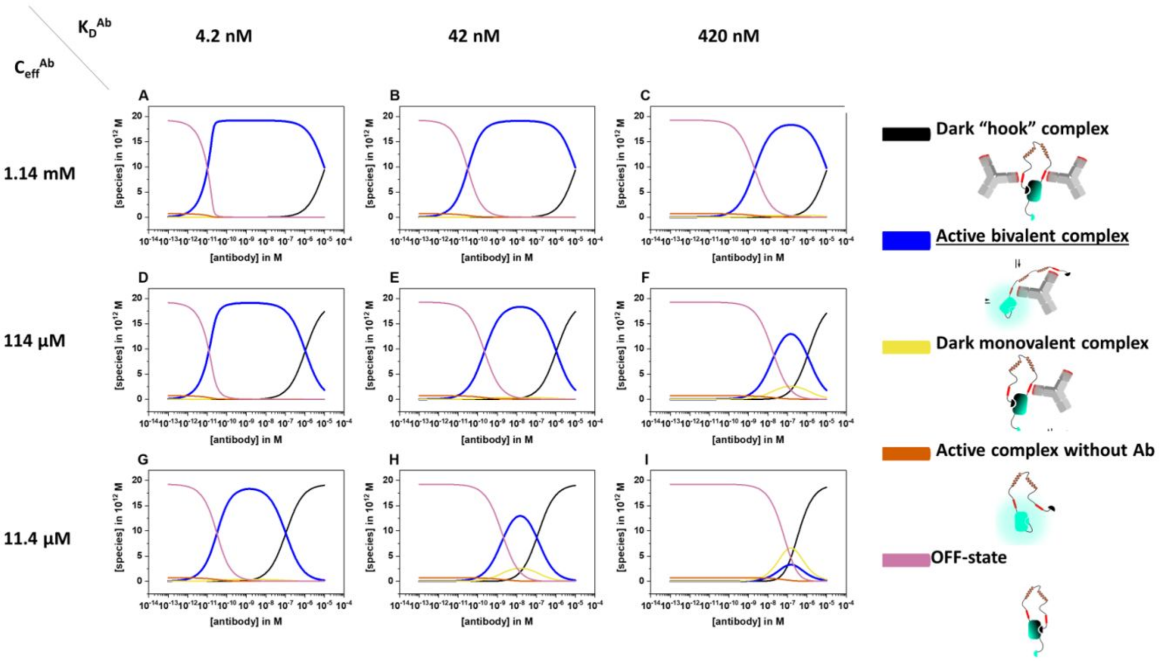

Figure S11 Simulations using the thermodynamic equilibrium model show that the fraction of active bivalent complex is strongly dependent on the monovalent affinity of the epitopes, K_D^{Ab}. For lower K_D^{Ab}, more antibody is required to achieve an active, bivalent complex. For lower C_{eff}^{Ab}, the equilibrium shifts to the dark, monovalent complex, which in turn leads to a stronger hook effect. Sensor concentration was set at 20 pM. All other parameters (in M) were left as follows: $C_{eff}^{DarkBiT}$:0.00173, $K_D^{DarkBiT}$:2.6E-6, K_D^{SmBiT}:1.9E-4, C_{eff}^{SmBiT}:4.66E-3.

Peptide & Protein sequences

In the following, SmBiTs are highlighted in yellow, DarkBiTs are highlighted in purple, epitopes are highlighted with red font, LgBiT or NanoLuc in light blue, mNeongreen in green.

DarkBiT101 – N-terminal acetylated, C-terminal amidated. Additional small amino acids were added to resemble the adjacent linker amino-acids in the switch construct

SVTGYALFEKESG

SmBiT86 – N-terminal acetylated, C-terminal amidated

VSGWRLFKKIS

LgBiT

MVFTLEDFVGDWEQTAAYNLDQVLEQGGVSSLLQNLAVSVTPIQRIVRSGENALKIDIHVIIPYEGLSAD
QMAQIEEVFKVVYPVDDHHFKVILPYGTLVIDGVTPNMLNYFGRPYEGIAVFDGKKITVTGTLWNGNKII
DERLITPDGSMLFRVTINGTTSAWSHPQFEK*

Two component model system – receptor: LgBiT-DarkBiT101 (precisely, LgBiT-epitope-linker-epitope-DarkBiT101)

MGSSHHHHHHSSGLVPRGSHMVFTLEDFVGDWEQTAAYNLDQVLEQGGVSSLLQNLAVSVTPIQRIVR
SGENALKIDIHVIIPYEGLSADQMAQIEEVFKVVYPVDDHHFKVILPYGTLVIDGVTPNMLNYFGRPYEGI
AVFDGKKITVTGTLWNGNKIIDERLITPDGSMLFRVTINSSGGGTELDRWEKIRLRPGGSGGSGGSGGSG
GSGGSGAEAAAKEAAAKEAAAKEAAAKEAAAKEAAAKAGSGGSGGSGGSGGSGGSGAEAAAKEAAAKE
AAAKEAAAKEAAAKEAAAKAGSGGSGGSGGSGGSGGSGGELDRWEKIRLRPTSGGSCGGSVTGYALFEK
ESGGSGGSWSHPQFEK*

Two component model system – reporter: mNeongreen-SmBiT99 (the flexible C-terminus of mNeongreen was removed)

MGSSHHHHHHSSGLVPRGSHMVSKGEEDNMASLPATHELHIFGSINGVDFDMVGQGTGNPNDGYEEL
NLKSTKGDLQFSPWILVPHIGYGFHQYLPYPDGMSPFQAAMVDGSGYQVHRTMQFEDGASLTVNYRYT
YEGSHIKGEAQVKGTGFPADGPVMTNSLTAADWCRSKKTYPNDKTIISTFKWSYTTGNGKRYRSTART
TYTFAKPMAANYLKNQPMYVFRKTELKHSKTELNFKEWQKAFTGFGGSVTGYRLFEKISGTTSAWSHP
QFEK*

dark-LUMABS-HIV #1 (derived from HIV-NB-LUMABS-6)

MGSSHHHHHHSSGLVPRGSHMVTGYRLFEKESGSGGSGGSGGSGGSGGSGSGGSGGSGGSGGSGGSVFTL
EDFVGDWEQTAAYNLDQVLEQGGVSSLLQNLAVSVTPIQRIVRSGENALKIDIHVIIPYEGLSADQMAQIE
EVFKVVYPVDDHHFKVILPYGTLVIDGVTPNMLNYFGRPYEGIAVFDGKKITVTGTLWNGNKIIDERLIT
PDGSMLFRVTINSSGGGTELDRWEKIRLRPGGSGGSGGSGGSGGSGGSGAEAAAKEAAAKEAAAKEAAA

KEAAAKEAAAKAGSGGSGGSGGSGGSGGSGAEAAAKEAAAKEAAAKEAAAKEAAAKEAAAKAGSGGSG
GSGGSGGSGGSGGELDRWEKIRLRPTSGGSCGGSVTGYALFEKESGGSGGSWSHPQFEK*

dark-LUMABS-HIV #2 (derived from HIV-NB-LUMABS-7)

MGSSHHHHHHSSGLVPRGSHMVTGYRLFEKESGSGGSGGSGGSGGSGGSGSGGSGGSGGSGGSGGSVFTL
EDFVGDWEQTAAYNLDQVLEQGGVSSLLQNLAVSVTPIQRIVRSGENALKIDIHVIIPYEGLSADQMAQIE
EVFKVVYPVDDHHFKVILPYGTLVIDGVTPNMLNYFGRPYEGIAVFDGKKITVTGTLWNGNKIIDERLIT
PDGSMLFRVTINSSGGGTELDRWEKIRLRPGGSGGSGGSGGSGGSGGSGAEAAAKEAAAKEAAAKEAAA
KEAAAKEAAAKAGSGGSGGSGGSGGSGGSGAEAAAKEAAAKEAAAKEAAAKEAAAKEAAAKAGSGGSG
GSGGSGGSGGSGGELDRWEKIRLRPTSGGSCGGSVTGYALFEKISGGSGGSWSHPQFEK*

dark-LUMABS-HIV #3 (derived from HIV-NB-LUMABS-1)

MGSSHHHHHHSSGLVPRGSHMVTGYRLFEEILGSGGSGGSGGSGGSGGSGSGGSGGSGGSGGSGGSVFTL
EDFVGDWEQTAAYNLDQVLEQGGVSSLLQNLAVSVTPIQRIVRSGENALKIDIHVIIPYEGLSADQMAQIE
EVFKVVYPVDDHHFKVILPYGTLVIDGVTPNMLNYFGRPYEGIAVFDGKKITVTGTLWNGNKIIDERLIT
PDGSMLFRVTINSSGGGTELDRWEKIRLRPGGSGGSGGSGGSGGSGGSGAEAAAKEAAAKEAAAKEAAA
KEAAAKEAAAKAGSGGSGGSGGSGGSGGSGAEAAAKEAAAKEAAAKEAAAKEAAAKEAAAKAGSGGSG
GSGGSGGSGGSGGELDRWEKIRLRPTSGGSCGGSVTGYALFEKESGGSGGSWSHPQFEK*

dark-LUMABS-HIV #4 (derived from HIV-NB-LUMABS-4)

MGSSHHHHHHSSGLVPRGSHMVTGYRLFEEILGSGGSGGSGGSGGSGGSGSGGSGGSGGSGGSGGSVFTL
EDFVGDWEQTAAYNLDQVLEQGGVSSLLQNLAVSVTPIQRIVRSGENALKIDIHVIIPYEGLSADQMAQIE
EVFKVVYPVDDHHFKVILPYGTLVIDGVTPNMLNYFGRPYEGIAVFDGKKITVTGTLWNGNKIIDERLIT
PDGSMLFRVTINSSGGGTELDRWEKIRLRPGGSGGSGGSGGSGGSGGSGAEAAAKEAAAKEAAAKEAAA
KEAAAKEAAAKAGSGGSGGSGGSGGSGGSGAEAAAKEAAAKEAAAKEAAAKEAAAKEAAAKAGSGGSG
GSGGSGGSGGSGGELDRWEKIRLRPTSGGSCGGSVTGYALFEKISGGSGGSWSHPQFEK*

dark-LUMABS-CTX, without additional cysteine that was used in the NB-LUMABS system

MGSSHHHHHHSSGLVPRGSHMVTGYRLFEEILGSGGSGGSGGSGGSGGSGSGGSGGSGGSGGSGGSVFTL
EDFVGDWEQTAAYNLDQVLEQGGVSSLLQNLAVSVTPIQRIVRSGENALKIDIHVIIPYEGLSADQMAQIE
EVFKVVYPVDDHHFKVILPYGTLVIDGVTPNMLNYFGRPYEGIAVFDGKKITVTGTLWNGNKIIDERLIT
PDGSMLFRVTINSSGGGTCVFDLGTRRLRCGGSGGSGGSGGSGGSGGSGAEAAAKEAAAKEAAAKEAAA
KEAAAKEAAAKAGSGGSGGSGGSGGSGGSGAEAAAKEAAAKEAAAKEAAAKEAAAKEAAAKAGSGGSG
GSGGSGGSGGSGGCVFDLGTRRLRCTSGGSSGGSVTGYALFEKESGGSGGSWSHPQFEK*

dark-LUMABS-101F

MGSSHHHHHHSSGLVPRGSHMVTGYRLFEEILGSGGSGGSGGSGGSGGSGSGGSGGSGGSGGSGGSVFTL
EDFVGDWEQTAAYNLDQVLEQGGVSSLLQNLAVSVTPIQRIVRSGENALKIDIHVIIPYEGLSADQMAQIE
EVFKVVYPVDDHHFKVILPYGTLVIDGVTPNMLNYFGRPYEGIAVFDGKKITVTGTLWNGNKIIDERLIT
PDGSMLFRVTINSSGGGTSGPTCKITVRLNDRGIIKTVNTISGGSRRALEAIEEARQAGMDVTFDCGGELR
EQLEKVQEVVEKGGSGGSGGSGGSGGSGGSGAEAAAKEAAAKEAAAKEAAAKEAAAKEAAAKAGSGGS

GGSGGSGGSGGSGAEAAAKEAAAKEAAAKEAAAKEAAAKEAAAKAGSGGSGGSGGSGGSGGSGGSGPTC
KITVRLNDRGIIKTVNTISGGSRRALEAIEEARQAGMDVTFDCGGELREQLEKVQEVVEKTSGGSSGGSVT
GYALFEKESGGSGGSWSHPQFEK*

Green enhanced NanoLantern (GeNL); Calibrator luciferase (mNeongreen-NanoLuc, with shortened C and N-termini, respectively)

MGSSHHHHHHSSGLVPRGSHMVSKGEEDNMASLPATHELHIFGSINGVDFDMVGQGTGNPNDGYEEL
NLKSTKGDLQFSPWILVPHIGYGFHQYLPYPDGMSPFQAAMVDGSGYQVHRTMQFEDGASLTVNYRYT
YEGSHIKGEAQVKGTGFPADGPVMTNSLTAADWCRSKKTYPNDKTIISTFKWSYTTGNGKRYRSTART
TYTFAKPMAANYLKNQPMYVFRKTELKHSKTELNFKEWQKAFTGFEDFVGDWRQTAGYNLDQVLEQ
GGVSSLFQNLGVSVTPIQRIVLSGENGLKIDIHVIIPYEGLSGDQMGQIEKIFKVVYPVDDHHFKVILHYGT
LVIDGVTPNMIDYFGRPYEGIAVFDGKKITVTGTLWNGNKIIDERLINPDGSLLFRVTINGVTGWRLCERI
LAGTTSAWSHPQFEK*

References

1. Roth, T. L.; Milenkovic, L.; Scott, M. P. A rapid and simple method for DNA engineering using cycled ligation assembly. *PloS one* **2014,** *9* (9), e107329. DOI: 10.1371/journal.pone.0107329.
2. Kok, S. de; Stanton, L. H.; Slaby, T.; Durot, M.; Holmes, V. F.; Patel, K. G.; Platt, D.; Shapland, E. B.; Serber, Z.; Dean, J.; Newman, J. D.; Chandran, S. S. Rapid and reliable DNA assembly via ligase cycling reaction. *ACS synthetic biology* **2014,** *3* (2), 97–106. DOI: 10.1021/sb4001992.
3. Ni, Y.; Arts, R.; Merkx, M. Ratiometric Bioluminescent Sensor Proteins Based on Intramolecular Split Luciferase Complementation. *ACS sensors* **2019,** *4* (1), 20–25. DOI: 10.1021/acssensors.8b01381.
4. Yang, C.; Sesterhenn, F.; Bonet, J.; van Aalen, E. A.; Scheller, L.; Abriata, L. A.; Cramer, J. T.; Wen, X.; Rosset, S.; Georgeon, S.; Jardetzky, T.; Krey, T.; Fussenegger, M.; Merkx, M.; Correia, B. E. Bottom-up de novo design of functional proteins with complex structural features. *Nature chemical biology* **2021. DOI:** 10.1107/S0907444910007493.
5. van Rosmalen, M.; Ni, Y.; Vervoort, D. F. M.; Arts, R.; Ludwig, S. K. J.; Merkx, M. Dual-Color Bioluminescent Sensor Proteins for Therapeutic Drug Monitoring of Antitumor Antibodies. *Analytical chemistry* **2018,** *90* (5), 3592–3599. DOI: 10.1021/acs.analchem.8b00041.
6. Ni, Y.; Rosier, B. J. H. M.; van Aalen, E. A.; Hanckmann, E. T. L.; Biewenga, L.; Pistikou, A.-M. M.; Timmermans, B.; Vu, C.; Roos, S.; Arts, R.; Li, W.; Greef, T. F. A. de; van Borren, M. M. G. J.; van Kuppeveld, F. J. M.; Bosch, B.-J.; Merkx, M. A plug-and-play platform of ratiometric bioluminescent sensors for homogeneous immunoassays. *Nature communications* **2021,** *12* (1), 4586. DOI: 10.1038/s41467-021-24874-3.
7. Krishnamurthy, V. M.; Semetey, V.; Bracher, P. J.; Shen, N.; Whitesides, G. M. Dependence of effective molarity on linker length for an intramolecular protein-ligand system. *Journal of the American Chemical Society* **2007,** *129* (5), 1312–1320. DOI: 10.1021/ja066780e.
8. Kjaergaard, M.; Glavina, J.; Chemes, L. B. Predicting the effect of disordered linkers on effective concentrations and avidity with the “Ceff calculator” app. *Methods in enzymology* **2021,** *647*, 145–171. DOI: 10.1016/bs.mie.2020.09.012.
9. van Rosmalen, M.; Krom, M.; Merkx, M. Tuning the Flexibility of Glycine-Serine Linkers To Allow Rational Design of Multidomain Proteins. *Biochemistry* **2017,** *56* (50), 6565–6574. DOI: 10.1021/acs.biochem.7b00902.
10. Geertjens, N. H. J.; Vink, P. J. de; Wezeman, T.; Markvoort, A. J.; Brunsveld, L. *A general framework for straightforward model construction of multi-component thermodynamic equilibrium systems*, 2021. DOI: 10.1101/2021.11.18.469126.
11. Sachs, L. *Applied statistics*: *A handbook of techniques*; Springer series in statistics; Springer, 1982.
12. Motulsky, H. J.; Neubig, R. R. Analyzing binding data. *Current protocols in neuroscience* **2010,** *Chapter 7*, Unit 7.5. DOI: 10.1002/0471142301.ns0705s52.

13. Hulme, E. C.; Trevethick, M. A. Ligand binding assays at equilibrium: validation and interpretation. *British journal of pharmacology* **2010,** *161* (6), 1219–1237. DOI: 10.1111/j.1476-5381.2009.00604.x.
